# miRNA-431 Prevents Amyloid-β-Induced Synapse Loss in Neuronal Cell Culture Model of Alzheimer's Disease by Silencing Kremen1

**DOI:** 10.3389/fncel.2018.00087

**Published:** 2018-03-28

**Authors:** Sean P. Ross, Kelly E. Baker, Amanda Fisher, Lee Hoff, Elena S. Pak, Alexander K. Murashov

**Affiliations:** Department of Physiology, Brody School of Medicine, East Carolina University, Greenville, NC, United States

**Keywords:** miRNA, amyloid-β, Kremen1, Dkk1, synaptic loss, Alzheimer's disease, mouse model, cortico-hippocampal culture

## Abstract

Synapse loss is well regarded as the underlying cause for the progressive decline of memory function over the course of Alzheimer's disease (AD) development. Recent observations suggest that the accumulation of the Wnt antagonist Dickkopf-1 (Dkk1) in the AD brain plays a critical role in triggering synaptic degeneration. Mechanistically, Dkk1 cooperates with Kremen1 (Krm1), its transmembrane receptor, to block the Wnt/β-catenin signaling pathway. Here, we show that silencing Krm1 with miR-431 prevents amyloid-β-mediated synapse loss in cortico-hippocampal cultures isolated from triple transgenic 3xTg-AD mice. Exposure to AβDDL (an amyloid-β derived diffusive ligand) or Dkk1 reduced the number of pre- and post-synaptic puncta in primary neuronal cultures, while treatment with miR-431 prevented synapse loss. In addition, treatment with miR-431 also prevented neurite degeneration. Our findings demonstrate that miR-431 protects synapses and neurites from Aβ-toxicity in an AD cell culture model and may be a promising therapeutic target.

## Introduction

Alzheimer's disease (AD) is a debilitating neurodegenerative disease characterized by a memory deficit and the progressive decline in cognitive function. Synapse loss is the best pathological correlate of cognitive decline in AD, and mounting evidence suggests that AD is primarily a disease of synaptic dysfunction (Koffie et al., 2011). Soluble oligomeric forms of amyloid beta (Aβ), the peptide that aggregates to form senile plaques in the brain of AD patients, have been shown to be toxic to neuronal synapses both *in vitro* and *in vivo*. Aβ oligomers inhibit long-term potentiation (LTP) and facilitate long-term depression (LTD), electrophysiological correlates of memory formation. Furthermore, oligomeric Aβ has been shown to induce synapse loss and cognitive impairment in animals. The molecular underpinnings of these observations are now being elucidated, and may provide clear therapeutic targets for effectively treating the disease. Here, we review recent findings concerning AD pathogenesis with a particular focus on how Aβ impacts synapses. Increasing evidence supports a critical role for Aβ in the pathogenesis of sporadic as well as familial forms of AD (Lukiw, 2012). The Wnt/β-catenin signaling pathway, while critical for neural development, also plays an important role in normal brain function and in mediating Aβ toxicity in AD (Inestrosa et al., 2012). Wnt signaling prevents the Aβ oligomer-induced events neuronal cell death by preserving mitochondrial structure in hippocampal neurons (Arrázola et al., 2017) prevents Aβ-mediated synapse degeneration (Alvarez et al., 2004) and improves behavioral deficit in AD animal models (Vargas et al., 2014). In addition, recent observations revealed that the accumulation of Aβ in the brain causes marked increase in levels of the Wnt antagonist Dickkopf-1 (Dkk1) (Caricasole et al., 2004). Several lines of evidence indicate that Dkk1 may be a critical player in the mediation of synapse loss in AD (Purro et al., 2014). Using a transgenic mouse model that inducibly expresses Dkk1 in the hippocampus, it was recently demonstrated that Dkk1 triggers synapse loss, impairs long-term potentiation, and induces learning and memory deficits(Marzo et al., 2016). In addition, the application of Dkk1 neutralizing antibodies prevented Aβ-mediated loss of synapses in hippocampal neuronal cultures (Purro et al., 2012). While neutralizing antibodies is a useful approach in experimental settings, the potential for application in ambulatory and clinical settings are severely limited. It is therefore essential to investigate other methodological approaches, such as RNA interference, that could provide long-term silencing of the potential targets. We have previously shown that Kremen1 (Krm1), a transmembrane receptor for Dkk1 and an antagonist of Wnt signaling, is specifically targeted by miR-431, which was confirmed by a pull-down-assay, an RT-qPCR, and a luciferase assay (Wu and Murashov, 2013). In the current paper we ask if silencing Krm1 could protect synapses from Aβ toxicity.

In this study, we took advantage of 3xTg-AD mouse model of AD which contains three mutations associated with familial AD (APP Swedish, MAPT P301L, and PSEN1 M146V). These animals develop progressive neuropathology such as Aβ plaques and neurofibrillary tangles (Oddo et al., 2003). Aβ deposits in the cortex appear by 6 months of age while tau pathology becomes evident at 12 months. However, cognitive impairment such as a retention/retrieval deficit may be observed as early as 4 months of age (Billings et al., 2005). Learning deficits are observed in both spatial and contextual based paradigms such as Barnes maze and fear conditioning tasks by 6.5 months (Stover et al., 2015). In this study, we compared effects of miR-431 treatment on AβDDL-induced synapse loss in adult cortico-hippocampal cultures derived from 3- and 6-months old 3xTg-AD and WT mice.

## Materials and methods

### Animals

All animal experiments were conducted on 3xTg-AD (B6;129-Psen1tm1Mpm Tg(APPSwe,tauP301L)1Lfa/Mmjax) mice and control animals (101045 B6129SF2/J) and approved by the ECU IACUC. Primary neuronal cultures were derived from animals of both sexes at 3 and 6 months of age.

### Cortico-hippocampal culture

The protocol for adult cortico-hippocampal culture was adapted from Brewer et al. protocol (Brewer and Torricelli, 2007). Briefly, cortexes and hippocampi were dissected from 3 and 6 month old mice, snipped, dissociated with papain in HABG for 30 min at 30°C, then re-suspended in papain free HABG (Hybernate A, 1xB27, 0.5 mM Glutamax, Thermo Fisher Scientific, Waltham, MA). After trituration, the cortico-hippocampal neurons were separated via density gradient centrifugation using Opti-prep density gradient solution (Sigma, gradient was prepared according to Brewer et al. protocol; Brewer and Torricelli, 2007). The enriched neuronal fraction was re-suspended in a Neurobasal A/B27 solution that was supplemented with 10 ng/ml mouse PDGF and 5 ng/ml mouse FGF (Thermo Fisher Scientific). These fractions were then promptly plated on poly-D-lysine (Sigma, St. Louis, MO) coated coverslips contained in a 24 well plate. The cells were cultured for 3 weeks. After the first 4 days of incubation, approximately one third of the media was removed and replaced. This step was repeated once per week thereafter.

### AβDDL (Aβ-derived diffusible ligands)

AβDDLs were prepared as previously described (Fa et al., 2010). Briefly lyophilized β-amyloid (1-42) peptide (American Peptide Company, Inc., Sunnyvale, CA) was resuspended in ice cold 1,1,1,3,3,3-Hexafluoro-2-Propanol (HFIP), incubated for 2 h to allow for Aβ monomerization, concentrated under vacuum by using a SpeedVac centrifuge (800 g, room temperature) until a clear peptide film is observed at the bottom of the vials and then stored at −80°C. Before use for cell culture treatment, Aβ film was re-suspend by adding DMSO to obtain a concentration up to 5 μM Aβ1-42 and sonicated in the water bath for 10 min to ensure complete re-suspension.

### miRNA

Gain-of-function and loss-of-function experiments were performed with Ambion® Pre-miR™ miRNA Precursor Molecules and Ambion®Anti-miR™ miRNA inhibitors (Thermo Fisher Scientific). Following incubation, the cells were transfected with either 100 nM hsa-miR-431-5p mimic or miRNA inhibitor using Lipofectamine 2000 (Thermo Fisher Scientific) reagent according to the manufacturer's protocol. Control cells were incubated with equal amounts of the transfection reagent as a negative control. After 48 h, the cells were treated with 20 ng/ml Dkk1 (R&D Systems, Minneapolis, MN) or 5 μM of AβDDL for 3 h.

### Immunofluorescence and microscopy

Cortico-hippocampal cultures were rinsed with cold PBS and fixed with ice cold 4% paraformaldehyde in PBS for 5 min, washed 3 times with PBST, and blocked with 10.0% goat serum for 1 h at room temperature. After blocking, the serum was removed and the cells were incubated with primary antibodies Synapsin-1 (1:1,000) (Sigma) and PSD-95 (1:200) (Cell Signaling Technologies, Danvers, MA) overnight at 4°C. This was followed by incubation with secondary antibodies conjugates including FITC and Rhodamine (Jackson ImmunoResearch Laboratories, West Grove, PA). Cells were stained with DAPI (Sigma) to visualize nuclei. TUJ-1 (Covance Research Products, Inc., Denver, PA, USA) was used to visualize neuronal cells and their processes. Goat polyclonal antibodies were used against Krm1 (R&D Systems, Minneapolis, MN, USA). Slides were mounted using anti-fading medium Fluoro-Gel (Electron Microscopy Sciences, Hatfield, PA).

### Image collection and analysis

Images were acquired using an Olympus IX81 Inverted fluorescent microscope and CellSens Dimension software (Olympus America, Inc., Center Valley, PA). Approximately 15–20 images were collected per cover slip. Neurites of 10–15 neurons per each image were measured according to previously described protocol (Murashov et al., 2005). The length of the longest neurite for each neuron, as well as the number of neurite branches and number of puncta per neuron were determined using ImageJ software (NIH, Bethesda, MD). Neurite length was measured tracing using only in neurons, which were completely distinguishable from neighboring cells. Axon length was normalized to the average length of the control axons. Synaptic puncta were normalized by neurite length visualized with antibody staining against tubulin or MAP2. The number, size, and colocalization of puncta were measured and analyzed by One-way ANOVA. All analyzes were performed double blind.

### PCR

Analyses of miR-431, Dkk1, Krm1, and Wnt in the brain were performed according to routine lab protocols (Wu et al., 2012). Real-time PCR reactions were carried out using EXPRESS SYBER® GreenER™ qPCR SuperMix Universal (Thermo Fisher Scientific) in triplicates for each cDNA sample on Applied Biosystems 7500 Real-Time PCR System (Thermo Fisher Scientific). Primers specific for each miRNA and mRNA were obtained using Invitrogen (Thermo Fisher Scientific). As an internal control, primers for S12 were added for RNA template normalization, and the relative quantification of gene and miRNA expressions was calculated against S12 using a 2-ΔΔCT method. Gene-specific primer for miR-431: 5′-CAGGCCGTCATGCAAA-3′.

### Statistics

The results were expressed as mean ± standard error of the mean in graphic and text representations. The difference between groups were evaluated with *t*-tests and One-way ANOVA with *post-hoc* Tukey test using GraphPad Prism version 5 for Windows (GraphPad Software; San Diego, CA). A *p*-value of less than 0.05 was considered statistically significant. All PCR data were log transformed before statistical analyzes. The level of significance was set at *P* < 0.05.

## Results

### Changes in synaptic puncta and neurite length after treatments with AβDDL and Dkk1 in 3-month old 3xTg-AD and WT mice

**Figure 1:**
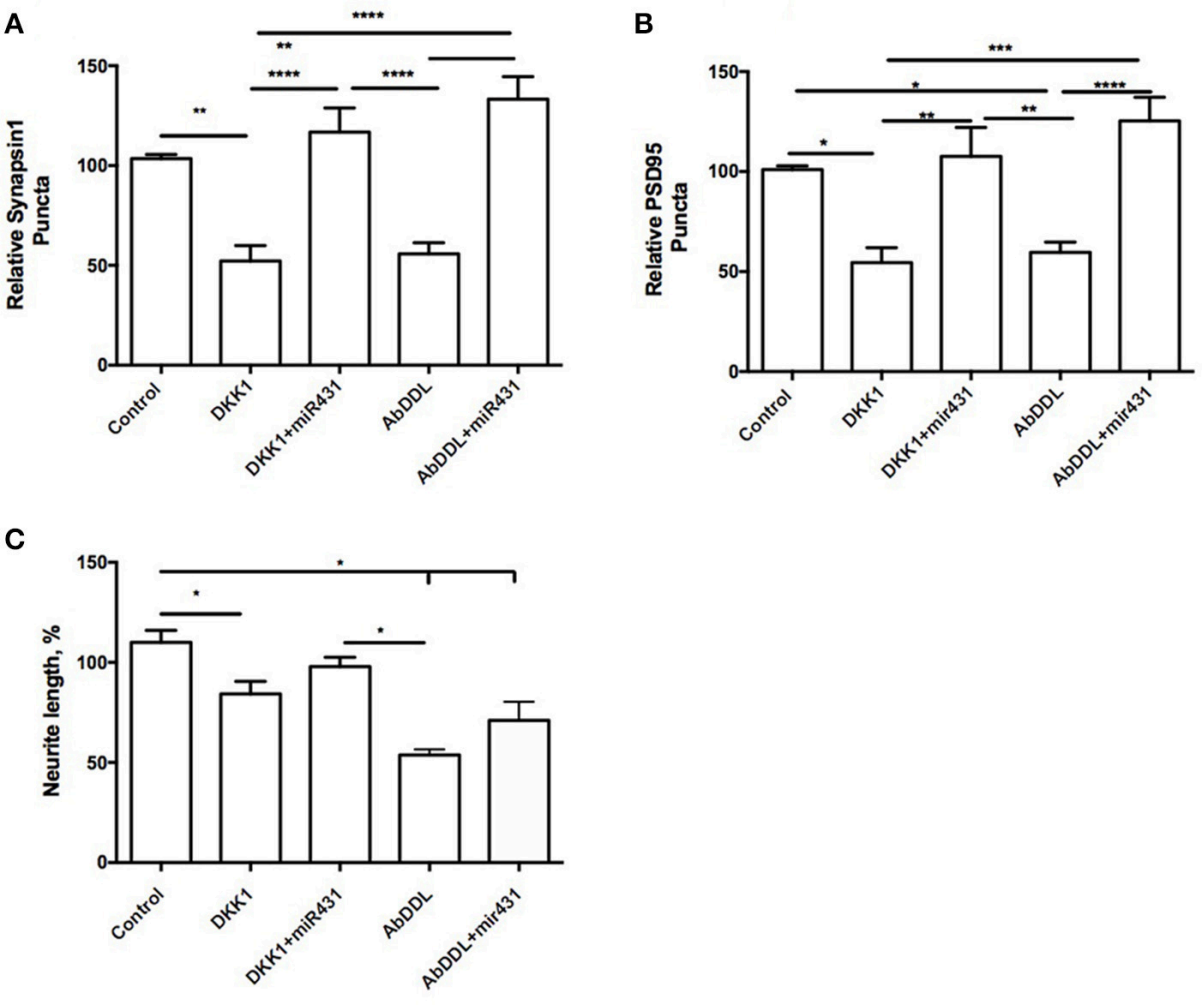
miR-431 prevents synapse loss in cortico-hippocampal cultures of 3-month 3xTg mice. Cultures of 3-month 3xTg mice transfected with miR-431 demonstrated significant increase in **(A)** pre-synaptic puncta and **(B)** post-synaptic puncta in comparison tp cultures treated with Dkk1, relative to control. **(C)** Average neurite length. ^*^p < 0.05, ^**^p < 0.005, ^***^p < 0.001, ^****^p < 0.0001.

Cortico-hippocampal cultures derived from 3 month old mice were transfected with either miR-431 or a negative miRNA mimic control. Forty-Eight hours later, the cultures were treated with AβDDL, Dkk1, or a vehicle. Twenty-Four hours after treatment, neuronal cultures were fixed, and non-direct immunofluorescence against synaptic proteins was used to examine pre- and post-synaptic puncta. The difference between groups were evaluated with *t*-tests and One-way ANOVA with *post-hoc* Tukey test. Cultures from 3xTg-AD mice transfected with miR-431 followed by treatment with Dkk1 showed a significantly higher number of presynaptic sites (116.7 ± 12.12, *n* = 9) in comparison to cultures treated with Dkk1 and negative miRNA mimic control (52.17 ± 7.788, *n* = 7, *p* < 0.0001) (Figure 1A). A similar dynamic for the synaptic puncta was observed between cultures treated with AβDDL and negative miRNA mimic control (55.76 ± 5.547, *n* = 17) and cultures treated with AβDDL and miRNA-431 (133.3 ± 11.21, *n* = 7, *p* < 0.0001) (Figures 1A, **6A–C**).

A number of post-synaptic sites in cultures treated with the negative miRNA mimic control and Dkk1 (54.47 ± 7.505) or AβDDL (59.65 ± 5.139, *n* = 19) were significantly lower in comparison to cultures transfected with miR-431 (Dkk1+miR-431: 107.6 ± 14.43, *n* = 8, *p* < 0.005; AβDDL+miR-431: *M* = 125.3 ± 11.87, *n* = 5, *p* < 0.001) (Figure 1B). There was no change in the amount of branching but significant decrease in neurite length after Dkk1 and AβDDL (Figure 1C).

**Figure 2:**
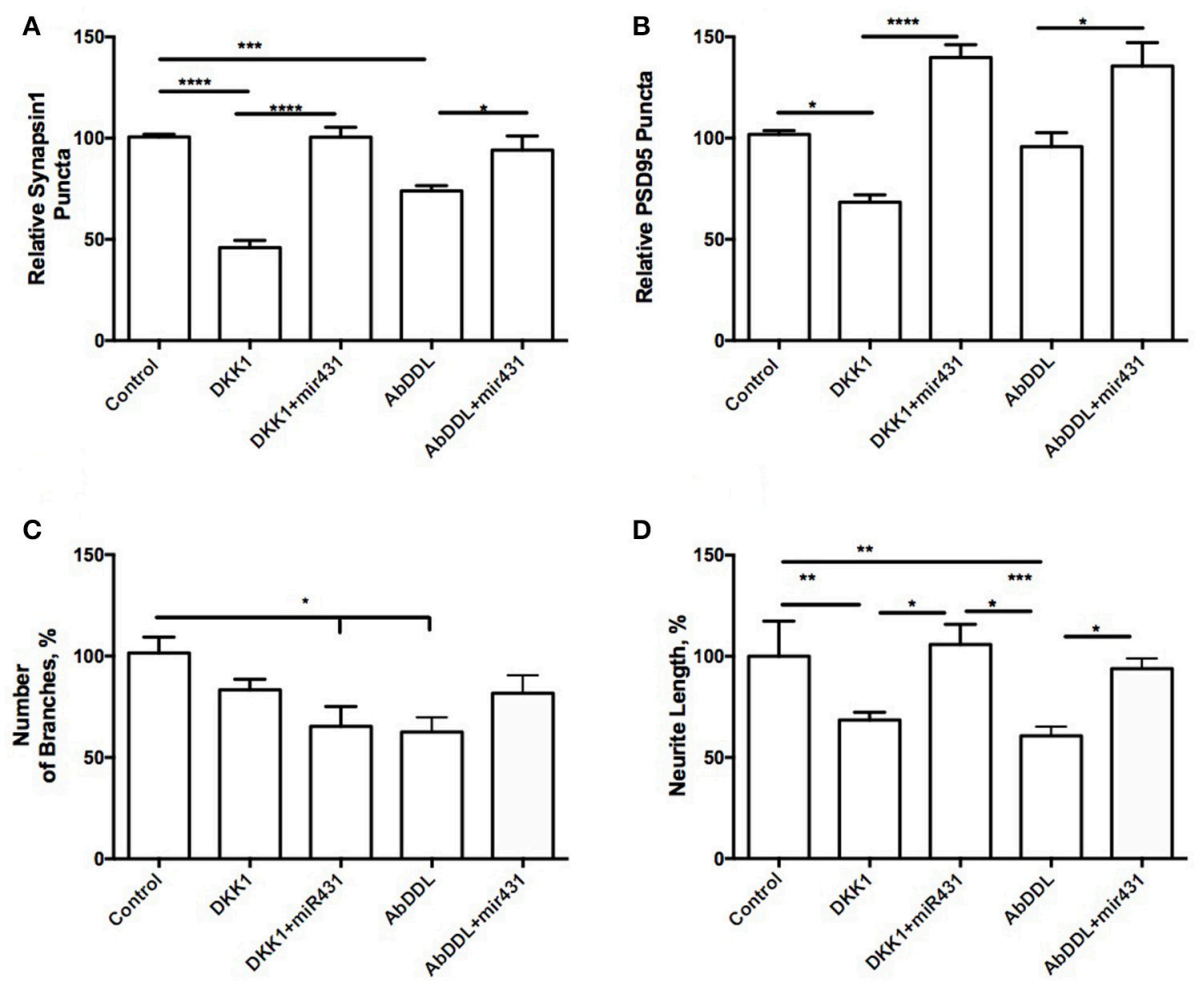
miR-431 prevents synapse loss in cortico-hippocampal cultures of 3-month WT mice. Cultures of 3-month WT mice transfected with miR-431 demonstrated significant recovery of **(A)** pre-synaptic puncta and **(B)** post-synaptic puncta in comparison to cultures treated with Dkk1. **(C)** Number of branches; **(D)** neurite length. ^*^p < 0.05, ^**^p < 0.005, ^***^p < 0.001, ^****^p < 0.0001.

Interestingly, cortico-hippocampal cultures derived from 3 month old WT mice showed very similar responses to the treatments. Cultures transfected with miR-431 followed by treatment with Dkk1 showed a significantly higher number of presynaptic sites (100.6 ± 4.851, *n* = 10) in comparison to cultures treated with Dkk1 and negative miRNA mimic control (45.96 ± 3.522, *n* = 18, *p* < 0.001) (Figure 2A). Similar results were observed between cultures treated with AβDDL and negative miRNA mimic control (73.94 ± 2.631, *n* = 22) and cultures treated with AβDDL and miR-431 (94.06 ± 6.970, *n* = 17, *p* < 0.05) (Figure 2A).

The number of post-synaptic sites in cultures treated with the negative miRNA mimic control and Dkk1 (71.32 ± 3.734, *n* = 16) or AβDDL (95.75 ± 6.883, *n* = 21) were significantly lower in comparison to cultures that also received treatment with miR-431 (Dkk1+miR-431: 139.8 ± 6.263, *n* = 9, *p* < 0.0001; AβDDL+miR-431: 141.2 ± 11.99, *n* = 12, *p* < 0.001) (Figure 2B). Neurite length in WT cultures was a more sensitive marker of the treatments with Dkk1, AβDDL and miR-431 (Figure 2D). We observed that treatment with miR-431 prevented neurite degeneration following Dkk1 and AβDDL treatments (Dkk1: 68.50 ± 3.816, *n* = 12; Dkk1+miRNA-431: 105.8 ± 10.02, *n* = 8, *p* < 0.005; AβDDL: 60.65 ± 4.620, *n* = 16; AβDDL+miRNA-431: 100.4 ± 5.931, *n* = 10, *p* < 0.001). However, there was significant change in the amount of branching after Dkk1+miRNA-431 and AβDDL (Figure 2C).

### Effect of miR-431 treatment on synaptic puncta and neurite length after exposure to AβDDL and Dkk1 in 6-month old 3xTg-AD and WT mice

**Figure 3:**
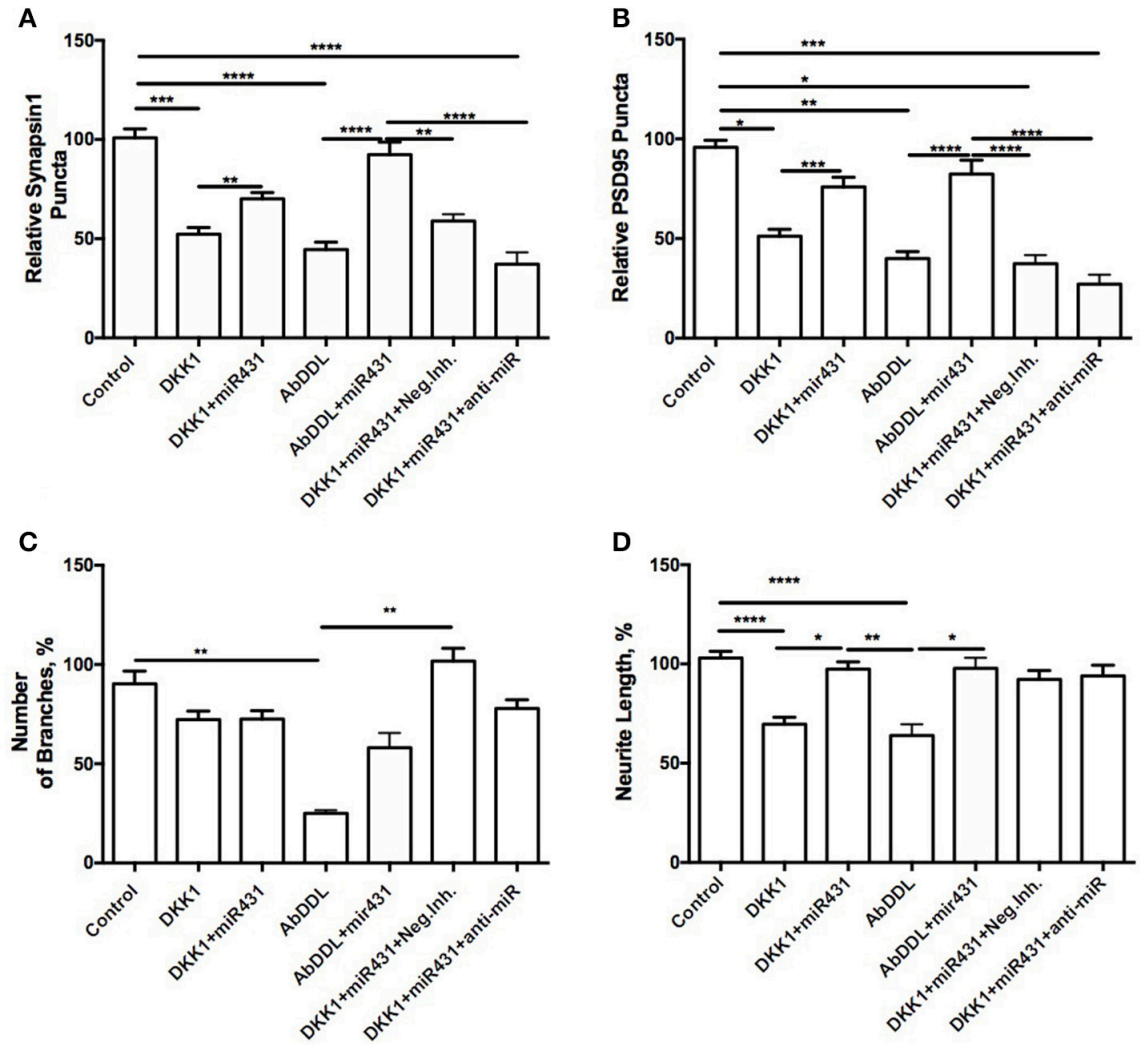
Prevention of synapse loss in cortico-hippocampal cultures of 6-month 3xTg mice. Cortico-hippocampal cultures of 6-month 3xTg mice transfected with miR-431 demonstrated significantly higher number of **(A)** pre-synaptic puncta and **(B)** post-synaptic puncta in comparison to cultures treated with Dkk1. **(C)** Number of branches; **(D)** neurite length. ^*^p < 0.05, ^**^p < 0.005, ^***^p < 0.001, ^****^p < 0.0001.

Cortico-hippocampal cultures derived from 6-month old 3xTg-AD and WT mice were transfected with either miR-431 or a negative miRNA mimic control. Forty-Eight hours later, the cultures were treated with AβDDL, Dkk1, or a vehicle. Twenty-Four hours after treatment, neuronal cultures were fixed, and non-direct immunofluorescence against synaptic proteins was used to examine pre- and post-synaptic puncta Cortico-hippocampal cultures from 3xTg-AD mice transfected with miR-431 prior to treatment with Dkk1 showed a significantly higher number of presynaptic sites (70.14 ± 3.097, *n* = 41) in comparison to cultures treated with Dkk1 and transfected with a negative miRNA mimic control (52.32 ± 3.343, *n* = 37, *p* < 0.005) (Figure 3A). Similar differences in synaptic puncta were observed between cultures treated with AβDDL and a negative microRNA mimic control (44.54 ± 3.747, *n* = 22) and cultures treated with AβDDL and miR-431 (92.43 ± 6.290, *n* = 16, *p* < 0.0001) (Figure 3A).

The number of post-synaptic puncta in cultures treated with the negative miRNA mimic control and Dkk1 (51.13 ± 3.477, *n* = 36) or AβDDL (39.93 ± 3.471, *n* = 22) were significantly lower in comparison to cultures transfected with miR-431 (Dkk1+miR-431: 75.92 ± 4.802, *n* = 25 *p* < 0.001; AβDDL+miR-431: 82.36 ± 6.969, *n* = 15, *p* < 0.0001) (Figure 3B).

Treatment with miR-431 prevented neurite degeneration following DKK1 and AβDDL treatments (Dkk1: 69.66 ± 3.474, *n* = 57; Dkk1+miR-431: 97.41 ± 3.648, *n* = 50 *p* < 0.0001; AβDDL: 63.97 ± 5.638, *n* = 14; AβDDL+miR-431: 97.81 ± 5.341, *n* = 14, *p* < 0.005) (Figures 3D, **6D–F**). At the same time, there was a significant change in the amount of branching after AβDDL (Figure 3C).

**Figure 4:**
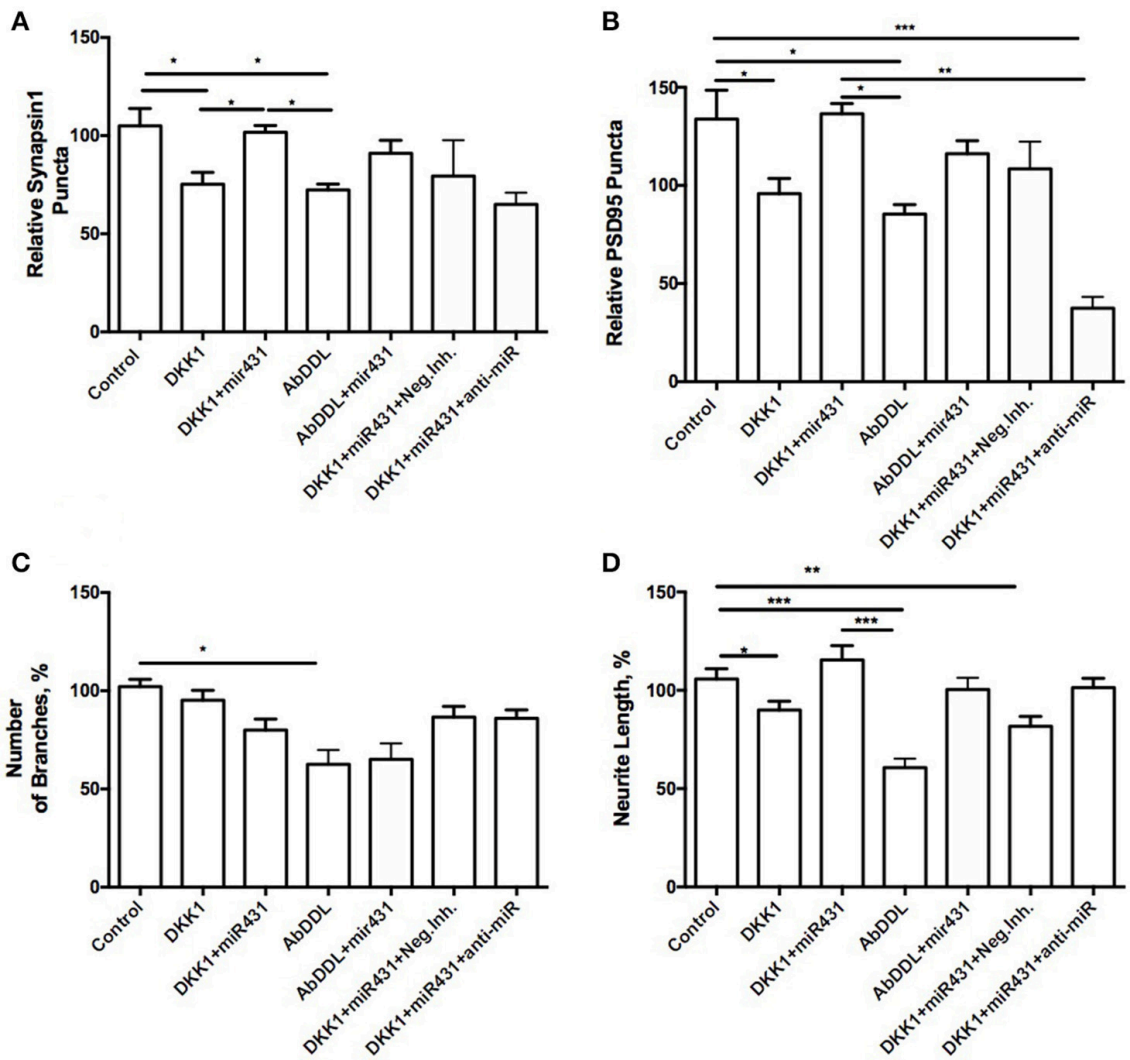
Prevention of synapse loss in cortico-hippocampal cultures of 6-month WT mice. Cortico-hippocampal cultures of 6-month WT transfected with miR-43. **(A)** pre-synaptic puncta and **(B)** post-synaptic puncta. **(C)** Number of branches, **(D)** neurite length. ^*^p < 0.05, ^**^p < 0.005, ^***^p < 0.001.

In WT cortico-hippocampal cultures derived from 6 month old mice, we observed dynamics in numbers of synaptic puncta very similar to cultures from 6-month 3xTg-AD animals. However, while the trends were the same, the observed changes failed to reach statistical significance. The number of presynaptic puncta sites in cultures treated with Dkk1 and transfected with miR-431 was 101.7 ± 3.535 (*n* = 15), while the number of presynaptic puncta sites in cultures treated with Dkk1 but not transfected with miR-431 was 80.06 ± 6.151 (*n* = 38) (Figure 4A). Similarly, the number of presynaptic sites in cultures treated with AβDDL but not transfected with miR-431 was 72.33 ± 2.983 (*n* = 23) vs. 90.98 ± 6.659 (*n* = 16) sites in cultures treated with AβDDL and transfected with miRNA-431(Figure 4A).

The number of post-synaptic puncta in cultures treated with Dkk1 (102.1 ± 8.381, *n* = 27) and AβDDL (85.44 ± 4.792, *n* = 19) were not significantly different from number of puncta in cultures that were transfected with miR-431 (Dkk1+miR-431: 136.5 ± 5.162, *n* = 11; AβDDL+miR-431: 116.1 ± 6.637, *n* = 9) (Figure 4B).

Significantly higher neurite length was observed in groups which received transfection with miR-431 and treatment with Dkk1 or AβDDL (Dkk1: 89.95 ± 4.522, *n* = 28; Dkk1+miR-431: 115.4 ± 7.309, *n* = 13, *p* < 0.05; AβDDL: 60.65 ± 4.620, *n* = 16; AβDDL+miR-431: 100.4 ± 5.931, *n* = 10, *p* < 0.001) (Figure 4D) vs. DKK1 or AβDDL treatments with a negative miRNA mimic control. Significant changes were observed in the amount of branching after AβDDL treatment (Figure 4C).

### Effect of AβDDL and miR-431 treatments on expression of Wnt signaling proteins in cortico-hippocampal neurons

We also asked how AβDDL treatment and transfection of the neuronal culture with miR-431 affects expression of Wnt signaling proteins including Dkk1, Krm1, β-catenin, and LRP6 at mRNA level. The experiment included the same treatment groups: control (vehicle treatment), AβDDL, miR-431, AβDDL+miR-431, AβDDL+miR-431+anti-miR miRNA inhibitor, AβDDL+miR-431+anti-miR negative control. Cortico-hippocampal cultures were transfected with miR-431 or a negative miRNA mimic control 48 h prior to treatment with AβDDL. Twenty-Four hours after treatment, neurons were collected and extracted RNA was used for qRT-PCR. The difference between groups were evaluated with *t*-tests and One-way ANOVA with *post-hoc* Tukey test.

**Figure 5:**
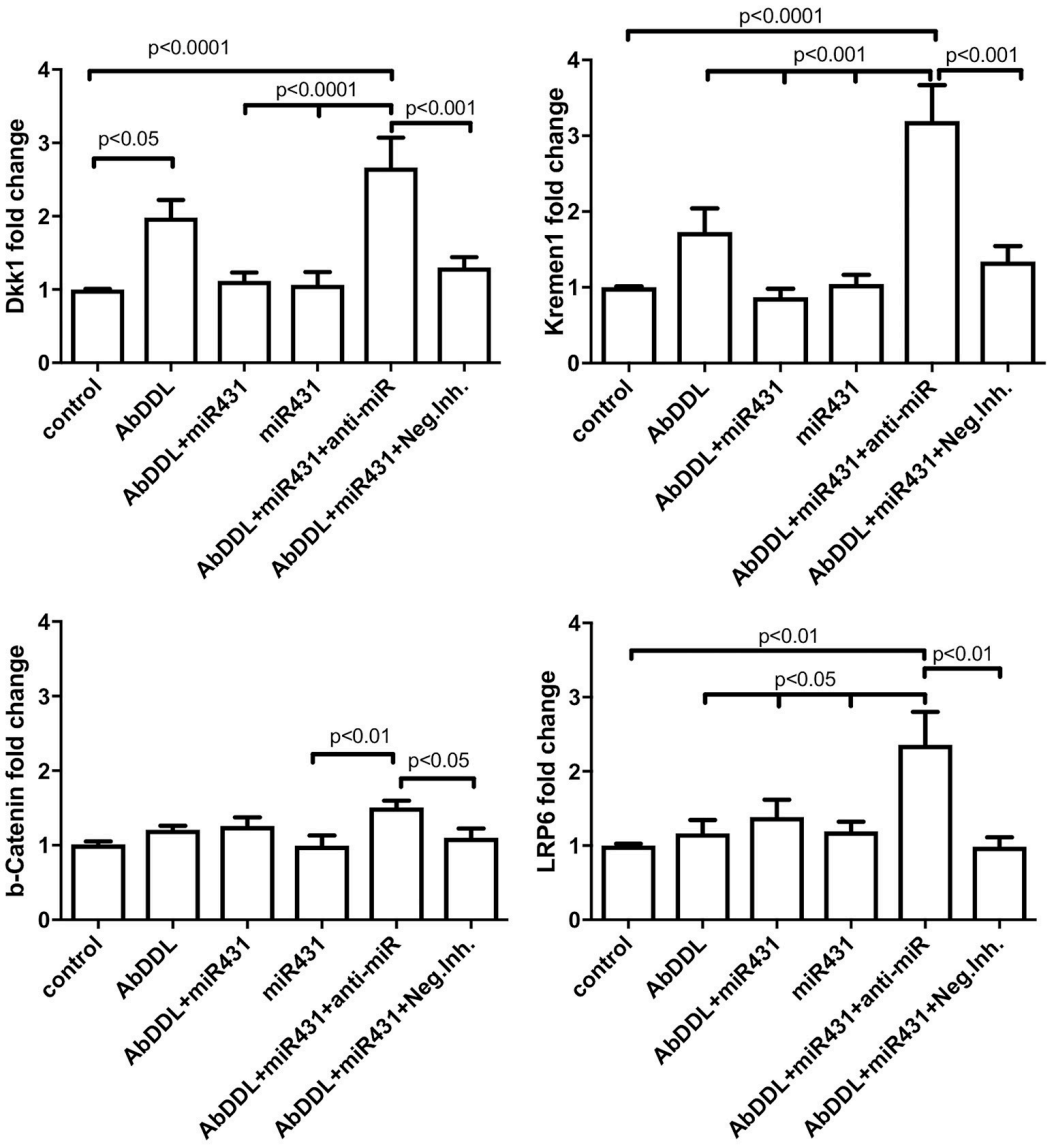
qRT-PCR analysis of Wnt signaling proteins after AβDDL and miR-431 treatments of cortico-hippocampal cultures of WT mice. Cortico-hippocampal cultures were transfected with miR-431 or a negative miRNA mimic control 48 h prior to treatment with AβDDL. 24 h after treatment neurons were collected and extracted RNA was used for qRT-PCR. Control cultures were untreated. As an internal control for PCR, primers for S12 were added for RNA template normalization. The relative quantifications of gene and miRNA expression were calculated against S12 by the ΔΔCt 2 method.

**Figure 6:**
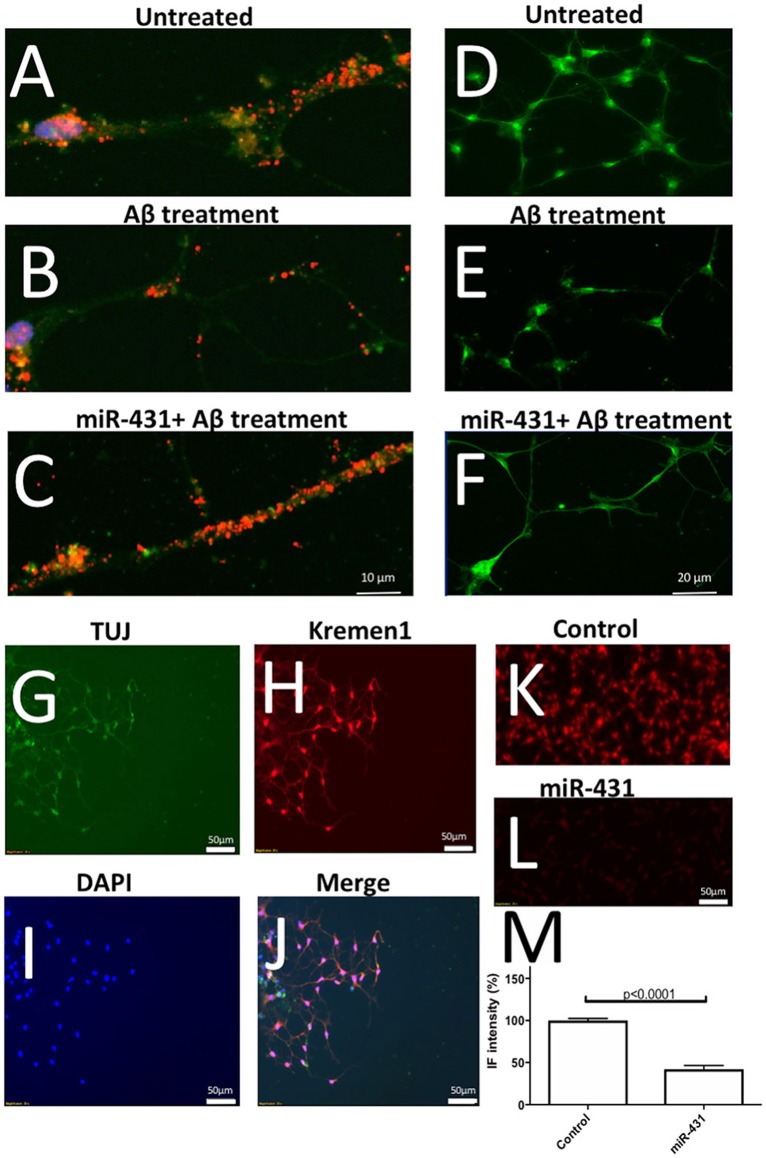
Representative immunofluorescent images of the performed experiments. **(A–C)** Illustrate effects of Aβ treatment and miR-431 transfection on synaptic puncta visualized with anti-PSD95 antibodies (Texas Red). DAPI (blue) was used to visualize nuclei and FITC (green) to visualize stating with Tuj antibodies against neuron-specific tubulin. The synaptic puncta is reduced after Aβ treatment **(B)** and preserved following miR-431 transection **(C)**. **(D–F)** Illustrate effects of Aβ treatment and miR-431 transfection on neurite length in cortico-hippocampal cultures visualized with Synapsin1 antibodies (FITC). The neurites degenerate after Aβ treatment **(E)** and protected following miR-431 transection **(F)**. **(G–J)** Shows triple-staining of WT cortico-hippocampal culture with TUJ antibodies (**G**, green), anti-Krm1 antibodies (**H**, red) and nuclear stain DAPI (**I**, blue). **(J)** Shows overlay of **(G–I)**. **(K**–**M)** Illustrate effect of miR-431 treatment of expression of Krm1. **(K)** Shows staining of untreated cortico-hippocampal culture with anti-Kremen antibodies. **(L)** Shows decreased expression of Krm1 after the culture was transfected with miR-431 for 48 h. **(M)** Graph shows relative reduction (percent change) in Krm1 fluorescent intensity after miR-431 treatment in comparison to untreated culture.

The experiment showed that AβDDL exposure induced a two-fold increase in Dkk1 and Krm1 (Figure 5). Interestingly, treatment with AβDDL+miR-431+anti-miR induced an even bigger 2.7–3 fold increase in Dkk1 and Krm1 levels. This increase was statistically significant against all other treatments groups except AβDDL. AβDDL exposure did not affect levels of β-catenin and LRP6. Intriguingly, anti-miR-treated cultures also showed significantly higher levels of Krm1, Dkk1, and LRP6 against the untreated control. At the immunocytochemistry level, we also observed that treatment with miR-431 significantly reduced expression of Krm1 (Figures 6G–M). These data indicate that miR-431 induced translational repression of Krm1 rather than mRNA degradation, which we observed in DRG culture in our previous publication (Wu and Murashov, 2013).

## Discussion

Our findings demonstrated that silencing Krm1 may be an effective way to protect synapses from Aβ-mediated toxicity. Interestingly, we observed that miR-431 treatment of cortico-hippocampal culture significantly reduced expression of Krm1 at the protein level but not mRNA level. These data indicate that miR-431 causes translational repression of Krm1 rather than mRNA degradation, which we observed in DRG culture in our previous publication (Wu and Murashov, 2013). The discrepancy between previous and current observations may be due to different cell types and different transfection reagents. In 2013 paper we used DRG primary culture while here we used cortico-hippocampal culture. We also used different transfection reagents which maybe contributed to discrepancy. In the previous paper we used LipofectamineTM LTX and Plus Reagent (Invitrogen) while in the current paper we used Lipofectamine 2000.

In cortico-hippocampal cultures derived from 3xTg-AD and WT mice, application of miR-431 prevented Aβ-induced synapse degeneration and promoted neurite outgrowth. Specifically, we observed a rescue of pre- and post-synaptic puncta after transfection with miR-431 in AβDDL and Dkk1 treated cultures. The statistically significant increase in the number of synaptic puncta was observed in all groups of animals except 6-month WT mice. These results suggest that miR-431 treatment may delay AβDDL-associated synapse degeneration in the 3xTg-AD mouse model. In addition to rescuing synaptic sites, miR-431 treatment also reversed the inhibitory effect of AβDDL and Dkk1 on neurite outgrowth in 3-month WT, 6-month old WT, and 6-month 3xTg-AD animals, which was evident by the preserved neurite length and number of branches. We have previously shown that peripheral nerve injury-induced miR-431 stimulates regenerative neurite growth in DRG sensory neurons by silencing Krm1, an antagonist of Wnt/beta-catenin signaling. Krm1 is a high-affinity transmembrane receptor for Dkk1. Dkk1 and Krm1 form a ternary complex which competes with Wnt for binding to the low-density lipoprotein receptor-related protein 6 (LRP6) and as a result destabilizes Wnt/β-catenin canonic signaling. While DKK1 is a prognostic marker for cancer progression (Kandimalla et al., 2017), it is also elevated in various neurological disorders including stroke, Parkinson's and AD (Shou et al., 2002; Caricasole et al., 2004; Scott and Brann, 2013; Purro et al., 2014). Studies in laboratory animals revealed that Dkk1 may promote glutamate toxicity in ischemic rodent brains while direct injection of Dkk1 into hippocampus causes apoptosis (reviewed; Scott and Brann, 2013). Interestingly, Dkk1 plays also a crucial role in early neural development. Dkk1 null mutation is embryonically lethal and results in missing anterior midbrain brain structures and disrupted limb patterning in affected mouse embryos (Mukhopadhyay et al., 2001). Paradoxically, during embryonic development, Dkk1 may play a role as a survival factor. Recent observation reported that Dkk1 protects cells from programed cell death during mouse neural plate formation (Causeret et al., 2016). The authors suggested that Krm1 is a *bona fide* receptor for Dkk1 with two independent signaling activities: Wnt inhibition through its extracellular domain in the presence of Dkk1 and apoptosis induction through its cytoplasmic domain in the absence of ligand (Causeret et al., 2016). However, several previous studies argued that Dkk1 may inhibit Wnt signaling via LRP6 independently from Krm proteins and that Krm have only modulatory role in specific cells (reviewed; MacDonald et al., 2009). Indeed, the physiological role of Krm proteins as Wnt antagonists in mammalian tissues remains far from being elucidated. Double knock-outs for *Krm1* and *Krm2* showed ectopic post-axial forelimb digits and expanded apical ectodermal ridges in mouse embryos (Ellwanger et al., 2008). While no embryonic lethality was observed in Krm1/Krm2 double mutants, the similarity in effects on limb patterning between the Krm1/Krm2 null mutant and Dkk1 heterozygous mutant phenotypes suggested that Krm proteins function together with Dkk1 in modulation of Wnt signaling during embryonic development (Ellwanger et al., 2008).

While the exact functions of Krm and Dkk1 in neural development remains unclear, recent observations show that both positive and negative modulation of Wnt/β-catenin signaling plays crucial role in developmental and physiological processes, including, tissue patterning, cancer cell-fate determination, and neurological disorders (Nakamura et al., 2008; Scott and Brann, 2013).

Several lines of evidence suggest a neuroprotective role for Wnt signaling in neurodegenerative disorders such as AD (reviewed in Inestrosa and Toledo, 2008). Evidently, Aβ binds to the extracellular cysteine-rich domain of the Wnt Frizzled receptor (Fz) inhibiting Wnt/β-catenin signaling. It has been also shown that Wnt pathway functions in the mature normal brain where it is associated with modulation of axonal remodeling, dendrite outgrowth, synaptic activity, and behavioral plasticity (Ortiz-Matamoros et al., 2013). In the hippocampus, several Wnt and Fz receptors have been found to be expressed in hippocampal neurons throughout life from the embryonic stages to adult (Cerpa et al., 2008). Several observations have also reported that Wnt/β-catenin pathway is important for hippocampal neurogenesis (Wu and Hen, 2013). Stimulation with extremely low-frequency electromagnetic fields (ELFEF) enhanced Wnt/β-catenin signaling in the brain which promoted neurogenesis and olfactory memory (Mastrodonato et al., 2018).

In conclusion, our results indicate that silencing of Krm1 can positively regulate neurite outgrowth and synapse formation in both WT and 3xTg-AD neuronal cells. Our finding that silencing Krm1 can protect synapses from AβDDL toxicity may provide new opportunities for therapeutic intervention in neurological disorders including AD. While efficient delivery of miRNA across blood-brain-barrier remains under intensive investigation, future advances may provide novel means for targeted silencing of Krm1 in the human brain.

## Author contributions

All authors listed, have made substantial, direct and intellectual contribution to the work, and approved it for publication.

### Conflict of interest statement

The authors declare that the research was conducted in the absence of any commercial or financial relationships that could be construed as a potential conflict of interest.
